# Implant-based versus autologous mastopexy after massive weight loss: Complications and patient satisfaction

**DOI:** 10.1016/j.jpra.2026.01.036

**Published:** 2026-02-04

**Authors:** Valentina Budini, Patris Agaraj, Francesca Maria Mancini, Erica Geors, Francesca Zanon, Vincenzo Vindigni, Franco Bassetto

**Affiliations:** Plastic Surgery, Padua University Hospital, Padua, Italy

**Keywords:** Breast implant, Breast surgery, MWL-patients, Autologous reconstruction, Mastopexy

## Abstract

Mastopexy surgery is among the procedures most requested by patients after massive weight loss. Many require a florid and voluminous breast, which is achieved thanks to the implantation of a breast prosthesis. However, mastopexy surgery with the prosthesis, especially with skin and subcutaneous tissues tested by significant weight loss, hides numerous risks and pitfalls. Is it, therefore, worth proposing this operation to post-bariatric patients? The study aims to answer this question by comparing two cohorts of post-bariatric patients undergoing mastopexy surgery with autologous tissues (74 women) and mastopexy with prosthesis implantation (64 women). Data demonstrate that patients who have implanted prostheses have a higher complication rate (*p* = 0.016), in particular, a more frequent recurrence of breast ptosis (*p* = 0.003). At the same time, patients with autologous reconstruction had a higher rate of wound dehiscence (*p* = 0.015). Despite this, patient satisfaction rates were comparable between the two surgeries (*p* = 0.684) The results were compared with the latest literature to have a more holistic understanding of the debate.

**Level of evidence IV:**

Evidence obtained from multiple time series with or without the intervention, such as case studies. Dramatic results in uncontrolled trials might also be regarded as this type of evidence.

## Introduction

Over the past 20 years, the number of people getting bariatric surgery has continuously increased. This has led to a growing number of massive weight loss patients (MWL). Bariatric surgery enhances metabolic health and general quality of life; however, the resultant loss of skin elasticity and subcutaneous support sometimes leads to soft-tissue abnormalities, especially in the breast region. Because of this, breast reshaping is becoming one of the most popular body contouring surgeries for women after bariatric surgery.^1^

The post-MWL breast presents a distinctive reconstructive challenge, marked by significant skin laxity, reduced dermal thickness, parenchymal atrophy, and absence of upper-pole fullness. These anatomical characteristics make patients susceptible to recurrent ptosis, contour abnormalities, and diminished long-term cosmetic stability, hence complicating surgical planning relative to non-MWL populations.

Implant-based mastopexy has become a popular way to restore breast volume and projection, especially in women who have lost a lot of glandular tissue. Numerous studies involving both aesthetic and post-bariatric cohorts have indicated favorable short-term aesthetic results and elevated patient satisfaction after surgery augmentation-mastopexy. Nonetheless, the utilization of breast implants in MWL patients is contentious, since the compromised soft-tissue envelope may elevate the likelihood of implant-associated problems, such as malposition, capsular contracture, recurrent ptosis, and reoperation.^2^^,^^3^

To reduce these dangers, other methods of autologous and implant support have been suggested, such as contouring the parenchyma, using auto-augmentation flaps, and using dermo-capsular slings. Autologous mastopexy approaches seek to restore breast volume and projection while minimizing implant-related complications; numerous authors have documented positive results in specific MWL cohorts. Nevertheless, autologous reconstruction may be constrained by inadequate remaining tissue, uncertain long-term volume retention, and elevated risks of wound-healing problems in patients with suboptimal skin quality.^4^^,^^5^

Even though there is more and more research on breast contouring following MWL,^6^^,^^7^^,^^4^^,^^8^ there aren’t many direct comparative studies that look at implant-based mastopexy against autologous mastopexy in patients who have had bariatric surgery. There is a notable absence of unanimity concerning complication profiles, reoperation rates, and patient satisfaction between these two methodologies when implemented in the distinct anatomical context of MWL patients.

The objective of this study was to compare postoperative complications, reoperation rates, and patient satisfaction among post-bariatric women undergoing mastopexy with or without breast implants, thereby offering evidence-based guidance for surgical decision-making in this complex patient demographic.

## Materials and methods

The study was conducted in accordance with the World Medical Association Declaration of Helsinki (June 1964) and subsequent amendments.

The manuscript was checked against the Strengthening the Reporting of Observational Studies in Epidemiology (STROBE) checklist (Supplemental Appendix).

We conducted a retrospective analysis of all breast reshaping surgeries performed at the Plastic Surgery unit of the Padua University Hospital, Italy, between January 2016 and May 2024. The study comprised adult female patients who had bariatric surgery, followed by weight loss, and had their breasts reshaped. The patients were divided into two groups: mastopexy with parenchyma remodeling and mastopexy with implants.

This study is therefore a retrospective cohort study comparing postbariatric patients undergoing mastopexy, with or without implants.

Every patient received nutritional and endocrinological evaluations and was previously treated by a bariatric surgery team.

Massive weight loss (MWL) was formally defined as a reduction in body mass index (ΔBMI) greater than 7 kg/m², in accordance with commonly adopted criteria in post-bariatric and body contouring literature, as this threshold is associated with clinically significant soft-tissue redundancy and skin laxity.

Stable weight for at least 6 months was required prior to breast reshaping surgery.

Exclusion criteria comprised: weight loss through diet and exercise alone, a history of breast cancer, uncontrolled comorbidities, serious mental illnesses, multiple body-contouring procedures in a single session, or secondary breast surgery. Following bariatric surgery, each patient was assessed in our clinic using the Pittsburgh Rating Scale to classify their breast ptosis, parenchymal descent, and asymmetry. While implant type and characteristics were customized, the diagnosis and surgical options were discussed, with a focus on final volume preferences.

Patients signed an informed consent form following thorough instruction on implant technique and risks, including LMWC lymphoma. Our university clinic complies with accepted practices for processing personal data, allowing the anonymized use of clinical information and images for teaching and research.

All mastopexy procedures, both implant-based and autologous, were performed by the same surgeon and as single-stage operations. No planned staged or delayed breast augmentation procedures were included in the study cohort.

In patients undergoing mastopexy with implants, after preoperative marking and pedicle elevation, the new nipple–areola complex position was defined. Round, high-projection, microtextured implants (100–400 cc) were placed in a subglandular pocket, as all patients demonstrated an upper-pole pinch test greater than 2 cm.^9^^,^ ^10^^,^ ^11^^,^ ^12^^,^^13^ Implant sizing was guided intraoperatively using sizers to optimize volume selection. A superior pedicle technique was systematically employed.^14^

Parenchymal reshaping was performed to improve implant coverage and lower-pole support. The inferior glandular tissue was preserved and internal sutures between the medial and lateral pillars were used to create a parenchymal sling beneath the implant, aiming to enhance upper-pole projection and reduce the risk of secondary ptosis. Skin closure was achieved according to the degree of redundancy, using either a periareolar or inverted-T pattern.

On the other hand, mastopexies in the parenchymal remodeling group employed either a superior or supero-inferior pedicle, depending on the degree of ptosis, tissue distribution, and skin redundancy.^15^^,^^16^ Access was either periareolar (round-block) or inverted-T (wise pattern), with inverted-T preferred for major excess. The upper pole projection was restored through autologous auto-augmentation by preserving, de-epithelializing, mobilizing, and rotating the inferior parenchyma on its vascular pedicle as a flap. It offered fullness and support by being anchored to the pectoralis fascia at the rib level. The skin was redraped and closed in layers, and mound projection was enhanced by additional pillar-to-pillar sutures.

Subcutaneous Enoxaparin at 40 mg daily was administered since the first day post-op, until the patient completed mobilization. We routinely used drains, left in place until they collected less than 50–70 cc per day.

Follow-up visits were scheduled at 7, 14, and 30 days, 3 and 6 months post-surgery. The retrospective study was primarily conducted using our clinic’s database, which is continually updated with patient information. Additionally, patients were contacted by phone for any further information needed.

Patient-reported outcomes were assessed using a numeric satisfaction scale ranging from 0 to 10, routinely adopted in our institution during the study period. Validated breast-specific PROMs, such as the BREAST-Q, were not systematically administered during the early years of the cohort and were therefore not included in the present analysis.

Data were extracted from the institutional electronic database and medical records; to minimize bias, a single-surgeon protocol and uniform postoperative care were applied. The following information was gathered: follow-up, age, BMI and pre/post bariatric weight, total weight loss, time to resume activities, and satisfaction (0–10). At 1, 3, and 6 months, the following complications were evaluated: capsular contracture > Baker II, recurrent ptosis, dog ears, scar changes/keloids, bleeding, seroma, DVT, dehiscence, infection, and implant displacement/asymmetry. Intraoperative modifications were separated from minor problems that had no effect on quality of life. Complications were defined according to standardized criteria:

Infection: erythema, swelling or purulent drainage requiring antibiotics within 30 days;

Seroma: clinically/US-confirmed collection requiring aspiration or drainage within 30 days;

Hematoma: blood collection requiring evacuation;

Wound dehiscence/necrosis: separation >1 cm requiring dressings or re-suturing;

Capsular contracture: Baker ≥ II;

Recurrent ptosis: NAC descent >2 cm below IMF or grade II–III ptosis within 6 months;

Implant malposition: clinical/imaging-confimed, requiring surgical correction;

Reoperation: any secondary operation related to the index procedure within 6 months;

Primary outcomes were postoperative complications within 6 months, while secondary outcomes were reoperation rate and patient satisfaction. IBM, Armonk, NY’s SPSS was used for the analyses. Frequencies were used to represent categorical data, and mean ± SD was used for continuous data. Kolmogorov-Smirnov was used to test for normality; chi-square was used to check for dichotomous data, Student’s t-test was used to check for normally distributed continuous data, and Mann-Whitney U was used to check for non-normal data. A significance level of *p* < 0.05 was established.

Continuous variables were analyzed as mean ± SD or median, according to distribution; no categorization was applied unless clinically justified (implant size groups).

To reduce selection bias, all eligible patients in the study period were included.

To reduce information bias we used consistent definitions and a single-surgeon protocol.

## Results

This study examined 138 patients who underwent mastopexy after bariatric surgery, divided into two groups: 74 patients (53.6%) without implants and 64 patients (46.4%) with implants.

Sample size was determined by all consecutive eligible cases available in our institutional database during the study period: a total of 193 patients were screened, out of which 55 were excluded (41 for multiple body contouring procedures in one single session, 9 for secondary procedures, 5 for incomplete data), thus leaving 138 patients for final analysis. The comparison between the two groups revealed significant differences in some clinical and postoperative variables, while others showed no statistically significant differences.

Statistically significant differences were found between the two groups in terms of age, weight loss and pre- surgery BMI. In the group without implants, patients had a higher age at surgery, lower weight loss and a higher pre- surgery BMI. The other patient characteristics were comparable between the two groups. (Table 1).

**Table 1:** Preoperative demographic and clinical characteristics.

	Overall 138 (100.0%)	No implant 74 (53.6%)	Implant 64 (46.4%)	*p* value
Patient age, mean ± SD	44.2 ± 11.1	46.8 ± 11.2	41.3 ± 10.3	**0.003**
Patient initial weight, mean ± SD	118 ± 21.8	114.4 ± 19.9	121.6 ± 23.5	0.053
Patient pre-surgery weight, mean ± SD	69.4 ± 10.1	69.7 ± 9.5	69.2 ± 10.8	0.759
Weight loss, mean ± SD	48.3 ± 18.8	44.7 ± 18.2	52.5 ± 18.7	**0.015**
Patient initial BMI, mean ± SD	43.0 ± 7.5	42.6 ± 7.4	43.4 ± 7.7	0.542
Patient pre-surgery BMI, mean ± SD	25.5 ± 3.3	26.1 ± 3.3	24.7 ± 3.3	**0.012**

Bold P value is lower than 0.05.

Multivariate logistic regression was used to assess the association between implant use and complications, adjusting for age, preoperative BMI, and total weight loss.

Statistically significant differences were found between the two groups in terms of complication rate and reoperation rate. (Table 2) In the group without implants, patients had a lower complication rate (27.0% vs 46.9%) and a lower reoperation rate (13.5% vs 34.4%).

**Table 2:** Postoperative outcomes.

		Overall 138 (100.0%)	No implant 74 (53.6%)	Implant 64 (46.4%)	*p* value
Time to normal activities, mean ± SD		4.0 ± 2.3	3.9 ± 2.5	4.1 ± 2.2	0.585
Satisfaction, mean ± SD		7.5 ± 2.6	7.4 ± 2.4	7.6 ± 2.1	0.684
Complications, *n* (%)	Yes	50 (36.2%)	20 (27.0%)	30 (46.9%)	**0.016**
	No	88 (63.7%)	54 (73.0%)	34 (53.1%)	
Reoperations, *n* (%)	Yes	32 (23.2%)	10 (13.5%)	22 (34.4%)	**0.004**
	No	106 (76.9%)	64 (86.5%)	42 (65.6%)	

Bold P value is lower than 0.05.

**Graph 1:**
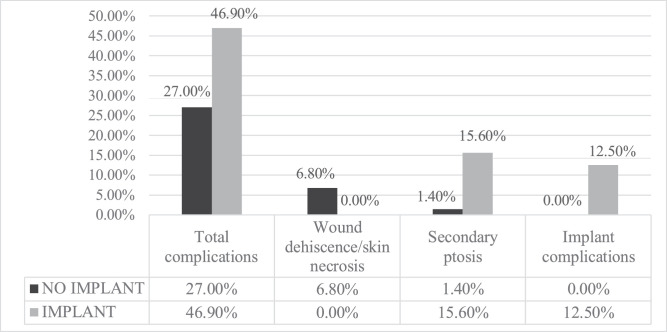
Complications.

Among complications, there were statistically significant differences between the two groups among the following aspects: the rate of wound dehiscence/necrosis, secondary ptosis, and implant complications (dislocation/contracture). (Table 3, Graph 1)

**Table 3:** Complications.

	Overall 138 (100.0%)	No implant 74 (53.6%)	Implant 64 (46.4%)	*p*
Bleeding *n* (%)	7 (5.1%)	4 (5.4%)	3 (4.7%)	1.000
Wound dehiscence/skin necrosis *n* (%)	7 (5.1%)	5 (6.8%)	0 (0.0%)	**0.015**
Infection *n* (%)	2 (1.4%)	1(1.4%)	1 (1.6%)	1.000
Secondary ptosis *n* (%)	11 (8.0%)	1 (1.4%)	10 (15.6%)	**0.003**
Asymmetry *n* (%)	11 (8.0%)	7 (9.5%)	4 (6.3%)	0.545
Seroma *n* (%)	1 (0.7%)	1 (1.4%)	0 (0.0%)	1.000
Dog ear *n* (%)	1 (0.7%)	1 (1.4%)	0 (0.0%)	1.000
TVP *n* (%)	1 (0.7%)	0 (0.0%)	1 (1.6%)	0.464
Diastasised or hypertrophic scars *n* (%)	3 (2.2%)	0 (0.0%)	3 (4.7%)	0.097
Implant complications (capsular contracture/implant displacement) *n* (%)	8 (5.8%)	0 (0.0%)	8 (12.5%)	**0.002**

**Fig. 1:**
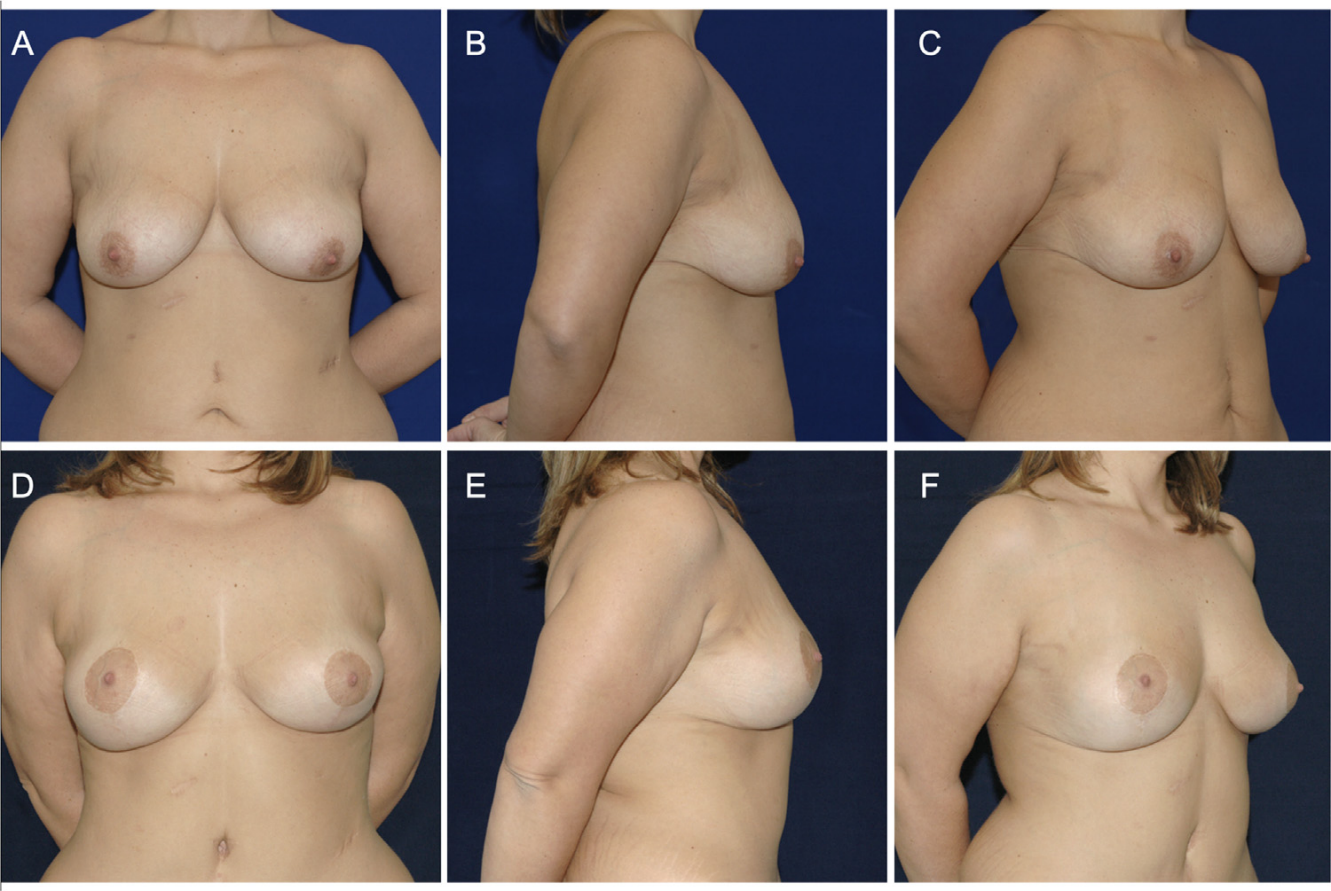
Clinical case of a 28-year-old patient who underwent correction of breast ptosis following bariatric surgery intervention, using a dermal-glandular auto prosthesis to restore shape and symmetry. Preoperative (A, B, C) and postoperative (D, E, F) photos.

In the group without implants, patients presented a higher rate of dehiscence/necrosis (6.8% vs 0.0%), a lower rate of secondary ptosis (1.4% vs 15.6%) and a lower rate of implant-related complications (0.0% vs 12.5%). (Figure 1).

**Fig. 2:**
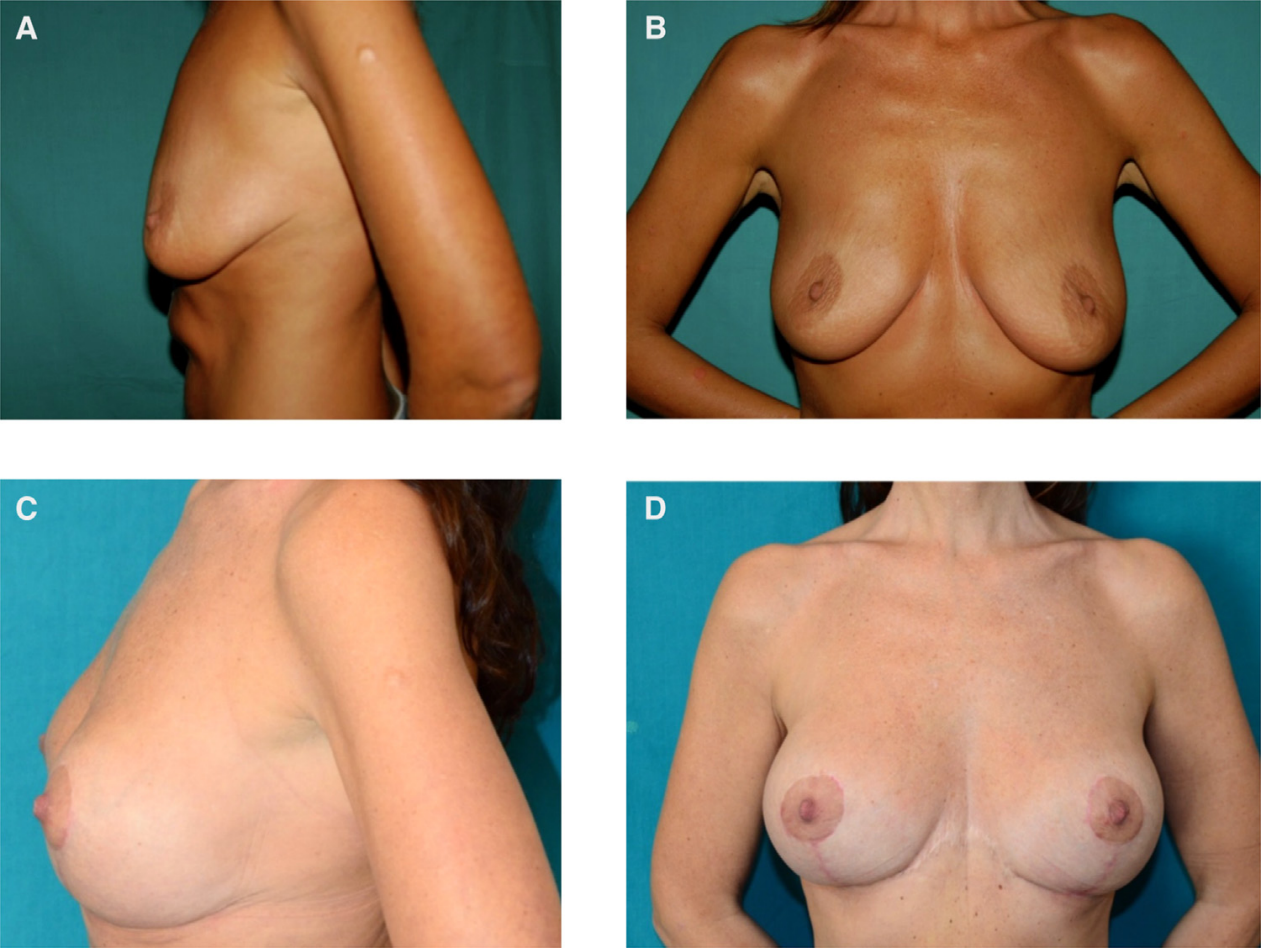
Clinical case of a 41-year-old patient treated for breast ptosis with mastopexy and 360 g polyurethane implants. Preoperative photos (A, B), postoperative outcomes (C, D).

Subgroup analysis by implant volume (<250, 251–300, >300 mL) was predefined to explore dose–response effects.

When evaluating complication rates by implant volume, the majority of patients received implants ≤300 mL, which were associated with notably high complication rates, 45.5% in the <250 mL group and 44% in the 251–300 mL group. The 301–350 mL subgroup showed the highest overall complication rate at 50%, along with a 43% reintervention rate. Interestingly, no complications or reinterventions were reported in patients with implants >350 mL. (Table 4)

**Table 4:** Complications based on implant volume.

PROSTHESIS VOLUME (mL)	Total	Percentage	Infection	Hematoma	Ptosis	Contracture	Asimmetry	Dystrophic scars	Complication rate	Reinter- ventions	Reinter- vention rate	Main cause of reintervention	Mean overall satisfaction (±SD)	Mean weeks to return to normal activities (±SD)
< 250	22	33.8%	0	1	2	1	4	2	45.5%	9	41%	Asimmetry^4^	7.23 ± 1.62	5.00 ± 2.59
251–300	25	38.5%	0	2	4	2	3	0	44%	4	16%	Ptosis^3^	7.76 ± 1.92	3.67 ± 2.20
301–350	14	21.5%	1	1	2	3	0	0	50%	6	43%	Contracture^3^	7.78 ± 2.54	3.80 ± 0.92
351–400	3	4.6%	0	0	0	0	0	0	0%	0	0%		9 ± 1.41	3.00 ± 2.83
> 400	1	1.6%	0	0	0	0	0	0	0%	0	0%		8	4.00

## Discussion

### Preoperative demographic and clinical characteristics

The average age was significantly different between the two groups (*p* = 0.03): the group without implants had an average age of 46.8 ± 11.2 years, whereas the group with implants had a lower average age of 41.3 ± 10.3 years. Albeit minimal, this difference may suggest a preference for implants among younger patients, possibly for aesthetic or volume restoration reasons.

Significant differences were also observed regarding weight loss after bariatric surgery (*p* = 0.015). Patients in the implant group lost an average of 52.5 ± 18.7 kg, compared to 44.7 ± 18.2 Kg in the non-implant group. This result may indicate that patients who experienced massive weight loss were more likely to undergo procedures with implants to address volume loss.

Preoperative BMI was also significantly different (*p* = 0.012): the non-implant group had an average BMI of 26.1 ± 3.3, compared to 24.7 ± 3.3 in the implant group. This result suggests that patients with implants tended to have a lower BMI before plastic surgery, possibly reflecting a greater need for aesthetic enhancement or volume restoration after more pronounced volume loss. Initial weight did not show statistically significant differences between the two groups (*p* = 0.092), with an average of 114.4 ± 19.9 kg in the non-implant group and 121.6 ± 23.5 Kg in the implant group. This result confirms that initial weight should not be a determining factor in the decision to use implants or just autologous tissues.

### Postoperative complications and satisfaction

After MWL, Mangialardi et al.^17^ examined autologous flaps as an alternative to implants, noting more natural outcomes and fewer implant-related risks like displacement and capsular contracture. This is corroborated by our data, which showed that the implant group experienced more complications (46.9%) than the non-implant group (27%; *p* = 0.016). Significant differences existed in minor complications: none of the cases with implants experienced wound dehiscence or skin necrosis, whereas 6.8% of the non-implant cases did (*p* = 0.015). With implants, secondary ptosis was more common (15.6% vs 1.4%; *p* = 0.003), which is indicative of tissue laxity and causes “waterfall deformity.” Furthermore, eight implant patients experienced prosthesis-specific issues (capsular contracture, displacement) (*p* = 0.02). These findings are consistent with those of Cogliandro et al.,^18^ who promoted dermo-capsular flaps as a means of reducing implant-related complications and offering structural support. Similarly, the time to resume normal activities was comparable between the two groups (*p* = 0.585), with an average of 3.9 ± 2.5 weeks in the non-implant group and 4.13 ± 2.2 weeks in the implant group. This result indicates that the prosthesis implant did not delay returning to daily activities.

Additionally, the implant group had a significantly higher reoperation rate (34.4%) than the non-implant group (13.5%) (*p* = 0.004), suggesting that patients with implants require more corrective or additional procedures, most likely as a result of implant-related complications and secondary ptosis corrections. This trend is in line with research by Sinik et al.,^19^ who observed that autologous procedures are becoming more popular for breast reconstruction after significant weight loss because they can reduce implant-related risks and provide safer long-term results. Our study revealed that patient satisfaction was similar in the two groups (7.4 ± 2.4 without implants vs 7.6 ± 2.1 with implants, *p* = 0.684), despite the higher complication rates linked to implants. This finding implies that even though implants carry a higher risk, they might still be able to satisfy patients’ aesthetic objectives. This conclusion is corroborated by Colwell et al.,^20^ who noted that obtaining the desired breast volume frequently determines patient satisfaction, even if doing so results in more complications. A significant percentage of patients (72.3%) received implants that were 300 mL or less, according to the analysis of complications by implant volume. Interestingly, these subgroups showed the highest rates of reinterventions and complications. The postoperative asymmetry (found in four cases) was the most common cause of the 41% reintervention rate and 45.5% overall complication rate in the <250 mL group. Ptosis was the most common reason for secondary surgical correction in the 251–300 mL subgroup, which showed a similar complication rate of 44%. Due mostly to the prevalence of capsular contracture, the 301–350 mL group had the highest rates of complications and reintervention (50% and 43%, respectively). These results show that in the massive weight loss (MWL) population, even relatively moderate implant volumes can be linked to significant postoperative morbidity, most likely as a result of decreased dermal integrity and parenchymal support.

Conversely, no complications or reinterventions were reported in patients receiving implants greater than 350 mL. However, this subgroup included only four individuals (three with implants between 351 and 400 mL and one exceeding 400 mL), making it statistically underpowered for meaningful interpretation. Therefore, the apparent absence of complications in this cohort should be approached with caution and cannot be generalized. Taken together, these data highlight the delicate balance between implant volume selection and complication risk in MWL patients. They reinforce the need for individualized surgical planning, with careful consideration of tissue characteristics and patient-specific anatomical and aesthetic goals, to improve long-term outcomes and reduce the likelihood of secondary procedures.

### Role of weight loss and skin quality

Surgical outcomes were also significantly influenced by weight loss and preoperative BMI. We found that the implant group lost more weight on average (52.5 ± 18.7 kg vs 44.7 ± 18.2 kg), and there was a significant difference in weight loss between the two groups (*p* = 0.015). This finding implies that patients who experienced greater weight loss might be more likely to choose implants in order to increase the volume of their breasts. Constantine et al.^21^ pointed out that the degree and mode of weight loss could have a big impact on the results of body contouring. Extreme weight loss frequently causes more noticeable skin laxity and possible deformities, so volume restoration is essential for patient satisfaction. Indeed, more severe skin laxity, decreased dermal thickness, and noticeable ptosis—factors that characterize a fundamentally different clinical scenario—are frequently associated with greater weight loss. These disorders raise the possibility of complications, complicate surgery, and may jeopardize long-term aesthetic stability. The study by Hany et al.^22^ also highlights the histological skin changes in post-bariatric patients, pointing out that surgical results can be impacted by the quality of the skin following significant weight loss. Following significant weight loss, patients who have less elastic skin may be more susceptible to complications like ptosis (*p* = 0.003 in our study), especially in the implant group (0.16% vs 0.01% in the non-implant group). These results bolster the case for customized surgical strategies depending on the degree of weight loss and the condition of the patient’s skin.

### Algorithmic approaches and technique selection

In line with Colwell et al.,^20^ who suggested taking age, weight loss, and skin quality into consideration when deciding between autologous tissue and implants, our results highlight the importance of customized surgical approaches. The Pittsburgh Rating Scale was developed by Pang et al.^23^ to grade post-MWL breast deformities and help choose a technique. A short-scar augmentation mastopexy was proposed by Salgarello et al.^24^ to reduce scarring and restore volume. This provides solutions that strike a balance between aesthetics and fewer complications, addressing problems observed in our implant group, where hypertrophic scars were more common (*p* = 0.09).

From the experience described in this study and from the comparative analysis with the results present in the literature, it emerges that in post-bariatric patients, the use of prostheses represents an additional risk of post- operative complications. The mass of a foreign body weighs on the patient’s tissues, which are already weakened by weight loss. In order to limit the risk of ptosis recurrence, reconstruction with autologous tissues should always be considered a first resort. However, there are cases in which volumetric filling can only be achieved with prostheses. In the latter, it is essential to study the case in depth and propose a prosthesis of the minimum volume necessary to restore harmonious breasts to the patient (Figure 2). Given the high risk of recurrent ptosis in this subset of patients and for the reasons previously outlined, the implant was placed in the prepectoral plane. Moreover, positioning the prosthesis in continuity with the overlying soft tissues is believed to yield a more natural breast contour and potentially reduce the rate of reoperation. Notably, in this cohort, capsular contracture was not a determining factor in preoperative planning, as patients did not present identifiable risk factors for its development. In this context, polyurethane-covered implants were selected to enhance adhesion between the surrounding tissues and the prosthesis surface, thereby minimizing the risk of secondary ptosis. Furthermore, new implant models can come to the surgeon’s aid: they guarantee a lower weight (for the same volume) thanks to the characteristics of the cohesive gel inside them. We believe that these new prostheses could constitute an excellent research starting point for the future of breast reconstruction in post-bariatric patients.

One limitation of this study is its retrospective design, which may introduce selection bias and limit the strength of causal inferences. To address this, we propose conducting a prospective randomized controlled trial in a similar patient population to validate our findings and provide higher-level evidence. Another limitation of this study is the relatively short follow-up period, which may primarily capture early complications rather than long-term aesthetic outcomes or implant-related issues. Longer-term follow-up is necessary to evaluate the durability of results, patient satisfaction, and potential late complications, and is planned for future analysis of this cohort.

Another limitation of this study is the absence of validated breast-specific PROMs, such as the BREAST-Q, which may limit the interpretability and external comparability of patient satisfaction outcomes. Future prospective studies from our institution will incorporate validated PROMs to provide a more comprehensive evaluation of patient-reported outcomes.

## Conclusion

In conclusion, comparing our results with the key studies on autologous flaps, implant-based techniques, and mastopexy after massive weight loss underscores the complexity of surgical decision-making in these patients. Autologous techniques, while avoiding implant-related complications, come with their peculiar risks and challenges. The use of implants, despite its higher complication rates, which indirectly also implies an increase in public spending, may offer quicker volume restoration and meet patient expectations for aesthetic outcomes. As a general rule, therefore, we prefer to avoid breast implants, due to the higher rates of reoperation and complications, alongside increased costs. Ultimately, what allows for the same level of postoperative patient satisfaction is the application of personalized approaches, taking into account the extent of weight loss, skin quality, and patient preferences, which are crucial for optimizing outcomes in post-bariatric mastopexy.

## Ethical approval

The displayed study was carried out with respect of high ethical standards. All the studies have been approved, when required, by the appropriate ethics committee and have, therefore, been performed in accordance and in conformity to the World Medical Association Declaration of Helsinki (June 1964) and subsequent amendments.

## Informed consent

All patients signed an informed consent for the procedures. For this type of study, formal consent is not required.

## Disclosure

None of the authors has a financial interest in any of the products, devices, or drugs mentioned in this manuscript.

## Funding

This research did not receive any specific grant from funding agencies in the public, commercial, or not-for-profit sectors. The study was entirely conducted with the institutional resources of the Plastic Surgery Unit, Padua University Hospital, without external financial support.

## Declaration of competing interest

The authors declare that they have no conflict of interest.

## Appendix Supplementary materials

Image, application 1
