# Supplementary material for: Implant-based versus autologous mastopexy after massive weight loss: Complications and patient satisfaction
Source: JPRAS Open. 2026 Feb 4;49:41–52. doi: 10.1016/j.jpra.2026.01.036 (PMC13080486; doi:10.1016/j.jpra.2026.01.036)
Supplement: Supplementary file 1 [file mmc1.docx]

**Supplementary Appendix**

**Supplementary Methods**

**Study Design**

This study is a retrospective cohort analysis conducted at the Plastic Surgery Unit of Padua University Hospital, Italy. All consecutive female patients undergoing mastopexy after massive weight loss (MWL) between January 2016 and May 2024 were screened for eligibility. Patients were divided into two cohorts according to surgical technique: mastopexy with implant placement and mastopexy with autologous parenchymal remodeling.

The study was designed and reported in accordance with the STROBE (Strengthening the Reporting of Observational Studies in Epidemiology) guidelines for observational studies.

**Definition of Massive Weight Loss**

Massive weight loss (MWL) was defined as a reduction in body mass index (ΔBMI) greater than 7 kg/m², a threshold commonly adopted in post-bariatric and body contouring literature and associated with clinically significant soft-tissue redundancy and skin laxity. Stable body weight for at least six months prior to breast reshaping surgery was required for inclusion.

**Eligibility Criteria**

**Inclusion criteria**

- Adult female patients (≥18 years)
- History of bariatric surgery
- Documented massive weight loss (ΔBMI >7 kg/m²)
- Stable weight for ≥6 months
- Primary mastopexy with or without breast implants

**Exclusion criteria**

- Weight loss achieved through diet and exercise alone
- History of breast cancer
- Uncontrolled medical comorbidities
- Severe psychiatric disorders
- Multiple body-contouring procedures performed in a single surgical session
- Secondary or revisional breast surgery
- Incomplete clinical data

**Preoperative Assessment**

All patients underwent standardized preoperative evaluation, including nutritional and endocrinological assessment and bariatric surgery follow-up. Breast morphology, ptosis severity, and asymmetry were classified using the Pittsburgh Rating Scale. Surgical planning was individualized based on breast anatomy, skin quality, parenchymal volume, and patient preferences regarding breast size and shape.

Informed consent included detailed discussion of surgical techniques, risks, and implant-related complications, including breast implant-associated anaplastic large cell lymphoma (BIA-ALCL).

**Surgical Technique**

**Implant-Based Mastopexy**

All implant-based procedures were performed as single-stage operations. After preoperative marking and elevation of a superior pedicle, the nipple–areola complex was repositioned. Round, high-projection, microtextured breast implants (100–400 cc) were placed in a subglandular pocket in patients with an upper-pole pinch test >2 cm.

Implant size selection was guided intraoperatively using sizers. Parenchymal reshaping was systematically performed to enhance implant coverage and lower-pole support. The inferior glandular tissue was preserved, and internal sutures between medial and lateral pillars were used to create a parenchymal sling beneath the implant, aiming to improve upper-pole projection and reduce secondary ptosis. Skin closure followed either a periareolar or inverted-T pattern, depending on skin excess.

**Autologous Mastopexy**

Autologous mastopexy procedures employed superior or supero-inferior pedicles based on ptosis severity and tissue distribution. Access was achieved through periareolar (round-block) or inverted-T (Wise pattern) incisions, with the latter preferred for severe skin redundancy.

Upper-pole projection was restored through auto-augmentation using inferior parenchymal flaps, which were de-epithelialized, mobilized, and rotated cranially while maintaining vascular supply. The flap was anchored to the pectoralis fascia to enhance projection and long-term stability. Additional pillar sutures were used to optimize breast shape and support.

**Postoperative Management and Follow-Up**

All patients received standardized postoperative care. Subcutaneous enoxaparin (40 mg daily) was administered starting on postoperative day one until full mobilization. Surgical drains were routinely used and removed when daily output was below 50–70 cc.

Scheduled follow-up visits occurred at 7, 14, and 30 days, and at 3 and 6 months postoperatively. Additional follow-up data were retrieved from institutional electronic records and, when necessary, through telephone interviews.

**Outcome Measures**

**Primary outcome**

- Postoperative complications occurring within 6 months

**Secondary outcomes**

- Reoperation rate within 6 months
- Patient satisfaction score (numeric scale 0–10)

Patient-reported satisfaction was assessed using a numeric rating scale routinely adopted in our institution during the study period. Validated PROMs such as the BREAST-Q were not systematically available and were therefore not included.

**Definitions of Complications**

Complications were defined according to standardized clinical criteria:

- **Infection:** erythema, swelling, or purulent drainage requiring antibiotics within 30 days
- **Seroma:** clinically or ultrasonographically confirmed fluid collection requiring aspiration or drainage within 30 days
- **Hematoma:** blood collection requiring surgical evacuation
- **Wound dehiscence/necrosis:** wound separation >1 cm requiring dressings or re-suturing
- **Capsular contracture:** Baker grade ≥ II
- **Recurrent ptosis:** nipple–areola complex descent >2 cm below the inframammary fold or grade II–III ptosis within 6 months
- **Implant malposition:** clinically or imaging-confirmed displacement requiring correction
- **Reoperation:** any secondary surgical procedure related to the index operation within 6 months

Minor intraoperative adjustments without impact on postoperative quality of life were not classified as complications.

**Statistical Analysis**

Statistical analyses were performed using IBM SPSS Statistics (IBM Corp., Armonk, NY). Continuous variables were reported as mean ± standard deviation or median, according to distribution. Normality was assessed using the Kolmogorov–Smirnov test.

Comparisons between groups were conducted using:

- Chi-square test for categorical variables
- Student’s t-test for normally distributed continuous variables
- Mann–Whitney U test for non-normally distributed variables

Multivariate logistic regression analysis was used to assess the association between implant use and postoperative complications, adjusting for age, preoperative BMI, and total weight loss. A p-value <0.05 was considered statistically significant.

**Supplementary Tables**

**Supplementary Table 1.** Extended demographic and preoperative characteristics
**Supplementary Table 2.** Detailed postoperative complication and reoperation rates
**Supplementary Table 3.** Breakdown of complications by type and surgical group
**Supplementary Table 4.** Subgroup analysis of complications according to implant volume

**Supplementary Figures**

**Supplementary Figure 1.** Flow diagram of patient selection and study inclusion
**Supplementary Figure 2.** Algorithm for surgical technique selection in MWL patients

**Reporting and Transparency**

This study complies with the STROBE checklist for observational studies. Ethical approval was obtained in accordance with institutional policies, and all patients provided informed consent for surgical procedures and anonymized data use. No external funding was received.
